# The CoGenT3 Study: Cognition and Gender Trends in Three American Generations

**DOI:** 10.1093/geroni/igaa057.2975

**Published:** 2020-12-16

**Authors:** Shana Stites

**Affiliations:** University of Pennsylvania, Philadelphia, Pennsylvania, United States

## Abstract

Many studies find gender differences in how older adults’ report on their memory, perform on cognitive testing, and manage functional impairments that can accompany cognitive impairment. Thus, understanding gender’s effects in aging and Alzheimer’s research is key for advancing methods to prevent, slow, manage, and diagnosis cognitive impairment. Our study, CoGenT3 – The study of Cognition and Gender in Three Generations – seeks to disambiguate the effects of gender on cognition in order to inform a conceptual model, guide innovations in measurement, and support future study. To accomplish this ambitious goal, we have gathered an interdisciplinary team with expertise in psychology, cognition, sexual and gender minorities, library science, measurement, quantitative methods, qualitative methods, and gender and women’s studies. The team benefits from the intersections of expertise in being able to build new research ideas, gain novel insights, and evaluate a wide-range of actions and re-actions but this novelty can also raise challenges.

